# Roles of LncRNAs in the Pathogenesis of Pulmonary Hypertension

**DOI:** 10.31083/j.rcm2506217

**Published:** 2024-06-17

**Authors:** Ting Liu, Shuanglan Xu, Jiao Yang, Xiqian Xing

**Affiliations:** ^1^Department of Pulmonary and Critical Care Medicine, Affiliated Hospital of Yunnan University, 650021 Kunming, Yunnan, China; ^2^Graduate School, Kunming Medical University, 650500 Kunming, Yunnan, China; ^3^Department of Pulmonary and Critical Care Medicine, First Affiliated Hospital of Kunming Medical University, 650032 Kunming, Yunnan, China

**Keywords:** pulmonary hypertension, long noncoding RNAs, pulmonary artery smooth muscle cells, pulmonary arterial endothelial cells, pulmonary adventitial fibroblasts, inflammatory and immune responses, right ventricle, vascular remodeling

## Abstract

Pulmonary hypertension (PH) is a persistently progressive, incurable, multifactorial associated fatal pulmonary vascular disease characterized by pulmonary vascular remodeling. Long noncoding RNAs (lncRNAs) are involved in regulating pathological processes such as pulmonary vasoconstriction, thickening, remodeling, and inflammatory cell infiltration in PH by acting on different cell types. Because of their differential expression in PH patients, as demonstrated by the observation that some lncRNAs are significantly upregulated while others are significantly downregulated in PH patients, lncRNAs are potentially useful biomarkers for assessing disease progression and diagnosis or prognosis in PH patients. This article provides an overview of the different mechanisms by which lncRNAs are involved in the pathogenesis of PH.

## 1. Introduction

Pulmonary hypertension (PH) is a syndrome characterized by structural or functional changes in the pulmonary vasculature with sex differences, often resulting in abnormal pulmonary arterial pressure and pulmonary vascular resistance [1]. Notably, female PH patients tend to exhibit better RV function and higher survival rates than men. It usually presents with enhanced proliferation of fibroblasts and pulmonary artery smooth muscle cells (PASMCs) and endothelial cell (EC) injury, leading to muscularization and thickening of small pulmonary arteries [2]. If left untreated, the disease progresses to right ventricular (RV) heart failure, ultimately leading to death [3]. Multiple cell types in the pulmonary arteries including vascular cells (smooth muscle cells, endothelial cells and fibroblasts) and inflammatory cells are abnormal in patients with PH [4]. Recently, with research on targeted drugs, the risk of deterioration and the prognosis of patients with pulmonary arterial hypertension have improved, but these treatments have not decreased the mortality rate. Therefore, further research on the mechanism of PH and the development of new drugs will help to further improve the overall prognosis of PH patients.

Long noncoding RNAs (lncRNAs) are transcripts that lack an open reading frame (ORF) and contain more than 200 nucleotides (nt) [5]. As functional molecules, lncRNAs can interact with RNA, DNA and proteins and participate in a variety of physiological or pathological processes, such as transcription processes; cell differentiation; chromosomal remodeling; stress responses; growth and development; and disease progression [6]. Previous studies have confirmed that lncRNAs are tightly associated with the development of PH, but specific mechanistic research is still in the exploratory stage. Therefore, this paper reviews the current literature on the effect of lncRNAs on PH to provide new insights into the clinical prediction, diagnosis and treatment of PH.

## 2. Overviews of lncRNAs

Based on previous evidence, more than 90% of the mammalian genome is transcribed as ncRNAs, with lncRNAs accounting for 80%–90% of the total ncRNA transcriptome. Recent studies have shown that these noncoding transcripts are not garbage or transcriptional noise and that they do have important biological functions, with regulatory lncRNAs a rapidly expanding type of such transcript [7].

### 2.1 Biogenesis of LncRNAs

The discovery of the RNA molecule began with the discovery of nucleic acids in 1869. Initially, lncRNAs were considered transcriptional “noise” because they lack efficient ORFs and have little to no protein-coding ability; however, with the launch of the Encyclopedia of DNA Elements project and the continuous development of high-throughput sequencing technologies, an increasing number of lncRNAs and their functions have been explored [8].

Based on spatial relationship with protein-coding genes, lncRNAs can be classified into six subtypes: sense lncRNAs, which contain overlapping exons of protein-coding genes transcribed from the same strand; antisense lncRNAs, which contain overlapping exons of protein-coding genes transcribed from the antisense strand; bidirectional lncRNAs, which are transcribed from mainly from the antisense strand; intronic lncRNAs, which are derived mainly from intronic regions of protein-coding genes; intergenic lncRNAs, which are derived mainly from the intergenic region of two protein-coding genes; and enhancer lncRNAs, which are derived mainly from the enhancer regions of protein-coding genes [9].

### 2.2 Biological Functions of lncRNAs

LncRNAs perform the following functions: (1) As transcriptional regulators, lncRNAs can function as cis-acting elements (in cis) or trans-acting elements (in trans) [6, 10]. (2) LncRNAs are involved in chromatin regulation. (3) LncRNAs play a role in nuclear organization. (4) LncRNAs are involved in posttranscriptional regulation, binding to specific proteins and forming specific lncRNA–protein (lncRNP) complexes in turn leading to changes in mRNA splicing and transcription. LncRNAs also pair with other RNAs to recruit protein complexes and can act as microRNA (miRNA) “sponges”. Some lncRNAs containing miRNA complementary sites can act as competing endogenous RNAs (ceRNAs) to control gene expression, thus reducing the targeting of mRNAs by miRNAs, which is particularly important for controlling the subsequent expression of the target mRNA. (5) Many lncRNAs are localized to specific organelles and can thus also regulate organelle function [11, 12, 13].

### 2.3 Methods for Studying lncRNAs

**Fig. 1.**
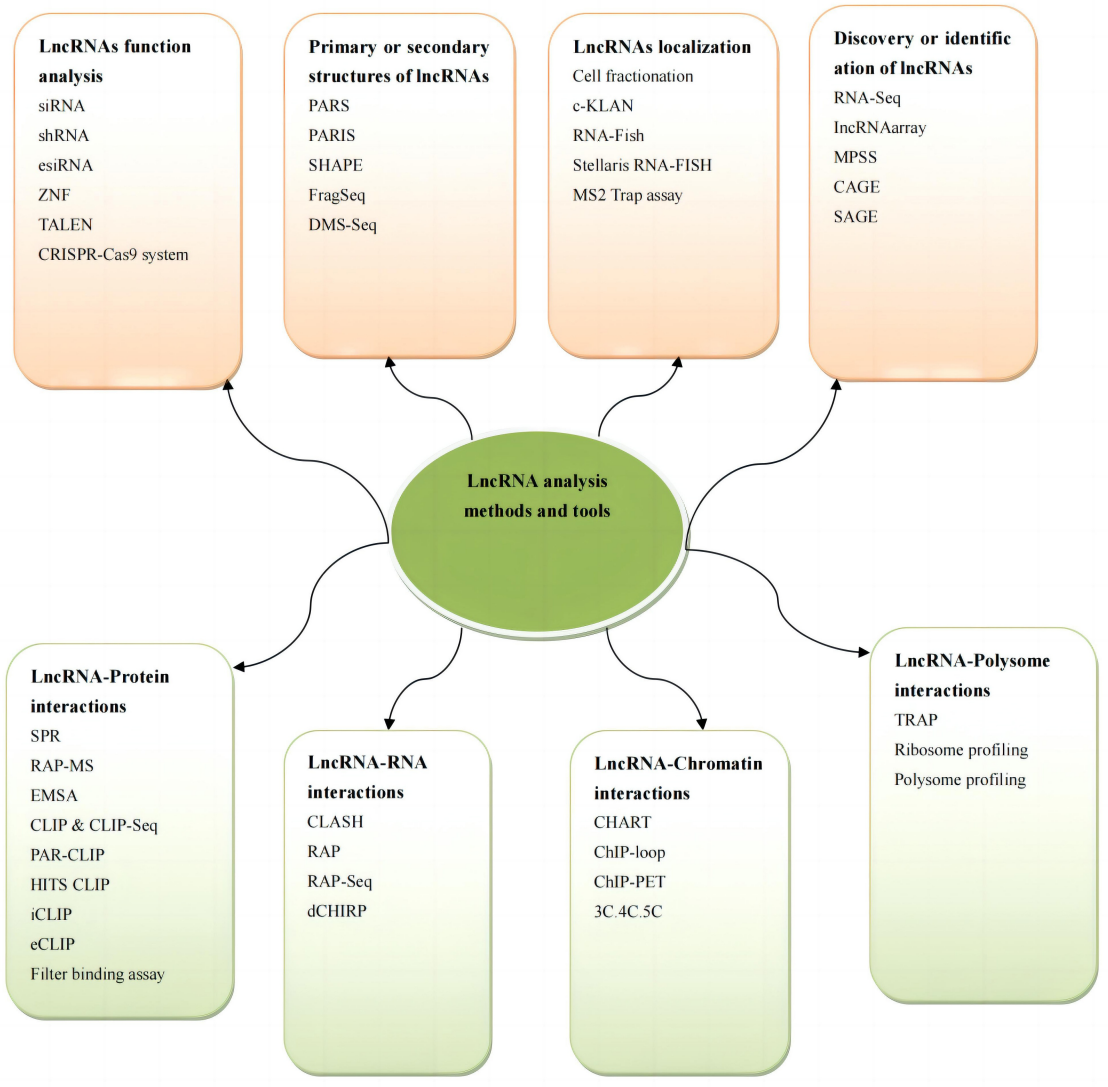
**Methods for studying lncRNAs**. ZNF, zinc-finger nuclease; TALEN, transcription activator like effector nuclease; PARS, RNA antisense purification; PARIS, psoralen analysis of RNA interactions and structures; SHAPE, selective $2^{\prime }$-hydroxylacylation analyzed by primer extension; DMS-Seq, dimethyl dulfate sequencing; c-KLAN, combined knockdown and localization analysis of noncoding RNAs; RNA-Fish, RNA fluorescence *in situ* hybridization; RNA-Seq, RNA sequencing; CAGE, cap analysis of gene expression; SAGE, serial analysis of gene expression; SPR, surface plasmon resonance; RAP-MS, RNA antisense purification with mass spectrometry; EMSA, electrophoretic mobility shift assay; CLIP, cross-linking immunoprecipitation; CLIP-Seq, cross-linking immunoprecipitation sequencing; PAR CLIP, photoactivable ribonucleoside enhanced CLIP; HITS CLIP, high-throughput sequencing of RNA isolated by crosslinking immunoprecipitation; iCLIP, individual-nucleotide resolution CLIP; eCLIP, enhanced CLIP; CLASH, crosslinking-ligation and sequencing ofhybrids; RAP, RNA antisense purification; RAP-Seq, RNA antisense purification sequencing; dCHIRP, domain-specifi chromatin isolation by RNA purification; CHART, capture hybridization analysis of RNA targets; TRAP, tagged RNA affinity purification; 3C, chromosome conformation capture; 4C, circular chromosome conformation capture; 5C, chromosome conformation capture carbon copy; lncRNAs, long noncoding RNAs; siRNA, small interfering RNA; shRNA, short hairpin RNA; esiRNA, endogenous small interfering RNAs; MPSS, massively parallel signature sequencing.

Compared with research on other RNAs, the study of lncRNAs is still in its infancy. Moreover, due to the complexity of lncRNA regulatory patterns and the tissue-specific and cell-specific expression of lncRNAs [14], identification and subsequent functional analysis of lncRNAs are extremely difficult. Previously, we determined the functions of lncRNAs in only a few different processes, including chromatin remodeling, genetic imprinting, splicing regulation, and transcriptional and translational regulation. However, with the advent of many new technologies, an increasing number of lncRNAs and their regulatory functions in humans are being discovered. The main approaches for studying lncRNAs include microarray analysis, RNA sequencing (RNA-seq) analysis, northern blotting, reverse transcription-polymerase chain reaction (RT‒PCR), and bioinformatics analysis, among others [15]. These new techniques have contributed greatly to the identification of diverse and dynamic lncRNAs, and the specific methods used in lncRNA research are shown in Fig. 1 [7, 16].

## 3. Molecular Mechanism of lncRNAs in PH

**Fig. 2.**
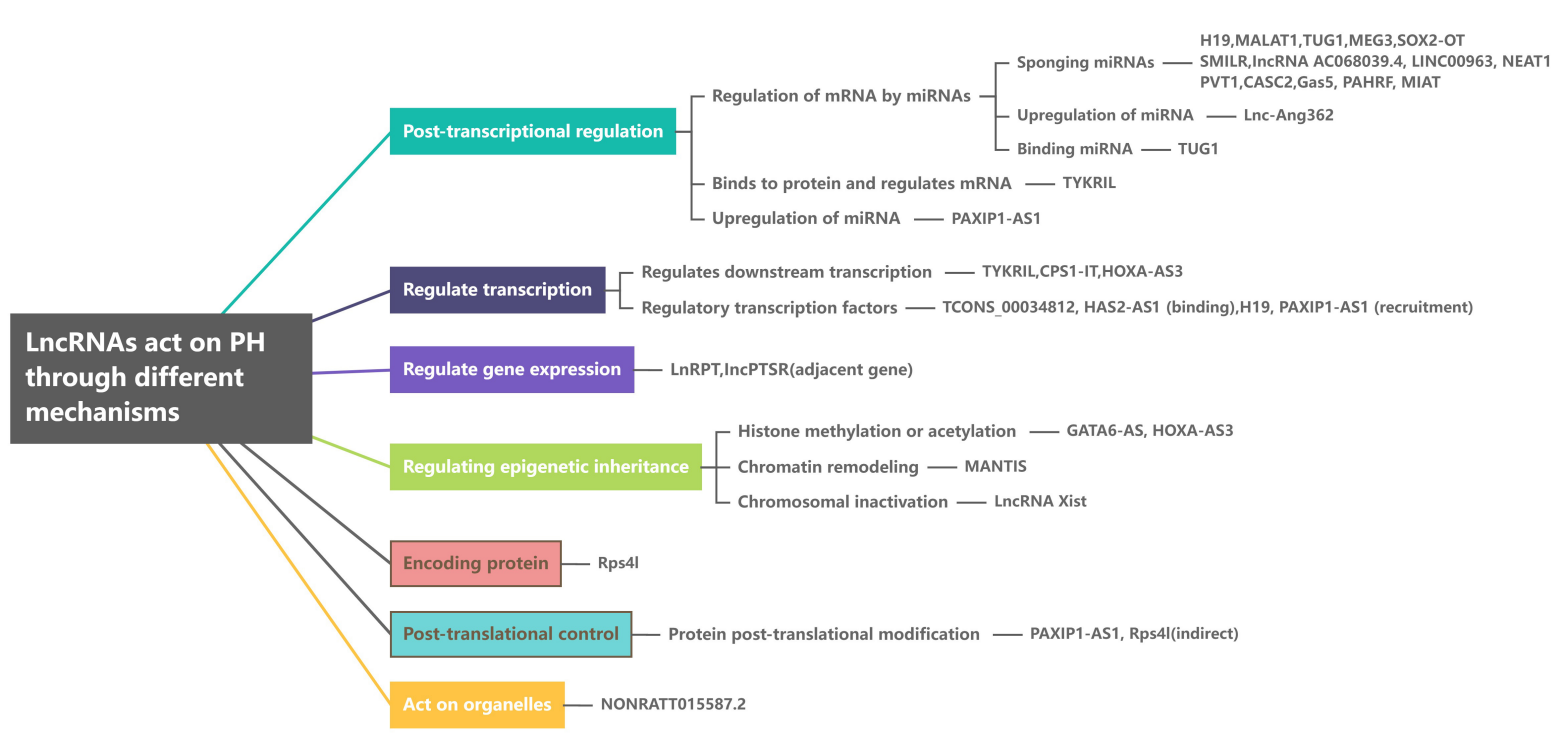
**LncRNAs act on PH through different mechanisms. **MALAT1, metastasis-associated lung adenocarcinoma transcript 1; TUG1, taurine-upregulated gene 1; MEG3, maternally expressed gene 3; SMILR, smooth muscle enriched long noncoding RNA; NEAT1, nuclear paraspeckle assembly transcript 1; PVT1, plasmocytoma variant translocation 1; CASC2, cancer susceptibility candidate gene 2; Gas5, growth arrest-specific 5; PAHRF, pulmonary arterial hypertension related factor; lnc-Ang362, lncRNA 362 regulated by angiotensin II; TYKRIL, tyrosine kinase recemtor inducing lncRNA; PAXIP1-AS1, PAXIP1 antisense RNA 1; CPS1-IT, CPS1 intron transcript 1; HOXA-AS3, HOXA cluster antisense RNA 3; LncPTSR, lncRNA neighboring the locus of ATPase plasma membrane ${\mathrm{Ca}}^{2+}$ β; Rps4l, lncRNA ribosomal protein S4-like; PH, pulmonary hypertension; MIAT, myocardial infarction-associated transcripts; LncRNAs, Long noncoding RNAs; SOX2-OT, lncRNA SOX2-overlapping transcript; LINC00963, long intergenic non-protein coding RNA 963; miRNA, microRNA; GATA6-AS, GATA6 antisense RNA 1; LnRPT, lncRNA regulated by PDGF and transforming growth factor β; mRNA, message RNA; HAS2-AS1, hyaluronan synthase 2 antisense 1.

LncRNAs are key molecules involved in the pathogenesis of PH by regulating gene expression at both the transcriptional and posttranscriptional levels [17]. In addition, lncRNAs can promote the proliferation of PASMCs by regulating organelle functions, for example, NONRATT015587.2 can act on mitochondria, and epigenetically regulate the endothelial cells function and the gender bias of PH, in which indirect regulation of post-transcriptional regulation of mRNA through miRNAs is its most commonly used mechanism, as detailed in Fig. 2.

Numerous lines of evidence suggest that lncRNAs play important regulatory roles in the pathogenesis and progression of PH and that they are involved in the pathogenesis of PH mainly through the regulation of pulmonary artery endothelial cell (PAEC) and pulmonary artery smooth muscle cell (PASMC) proliferation, apoptosis resistance, migration, and endothelial-mesenchymal transition (EndMT) [18]. In addition, a recent study showed that several lncRNAs also participate in the vascular inflammatory response in PH and are associated with the activation and release of various cellular inflammatory factors [19]. Some lncRNAs also regulate the phenotypic conversion of pulmonary adventitial fibroblasts (PAFs), which are involved in pulmonary vascular remodeling [20, 21].

### 3.1 LncRNAs and PASMC in PH

LncRNAs are implicated in the induction of different phenotypes in PASMCs, including phenotypic transitions involved in proliferation, apoptosis, migration, and cell cycle regulation [22]. The regulation of PASMCs is also the most widely studied aspect of this process. This review presents a summary of 27 lncRNAs associated with phenotypic changes in PASMCs. Among these 27 lncRNAs, 17 promoted and 10 suppressed the proliferation and migration of PASMCs respectively (Table 1, Ref. [23, 24, 25, 26, 27, 28, 29, 30, 31, 32, 33, 34, 35, 36, 37, 38, 39, 40, 41] and Table 2, Ref. [42, 43, 44, 45, 46, 47, 48, 49, 50, 51, 52, 53, 54, 55, 56]).

**Table 1.** **LncRNAs that promote the proliferation and migration of PASMCs**.

LncRNA	Regul-ation	Species	Models established	Targets	Cells	Functions	Ref
H19	↑	Mice, rats	MCT-induced	PDGF-BB/let-7b/AT1R	PASMCs	Promotes proliferation	[23]
MALAT1	↑	Humans	Cell culture	KLF5/miR-124-3p.1	hPASMCs	Hyperactivates the cell cycle and increases proliferation and migration	[24]
				miRNA/TLR4		Promotes proliferation and migration and inhibits apoptosis	[25]
Lnc-Ang362	↑	Humans	Cell culture	miR-221, miR-222/NF-kB	hPASMCs	Promotes proliferation and migration	[26]
PAXIP1-AS1	↑	Humans	Cell culture	Paxillin	hPASMCs	Promotes proliferation	[27]
		Rats	MCT-induced	ETS1/WIPF1/RhoA		Promotes cell viability and migration	[28]
TUG1	↑	Mice	Hypoxia -induced	miR-374c/Foxc1, notch	PASMCs	Promotes proliferation, migration and cell cycle progression	[29]
HOXA-AS3	↑	Mice	MCT-induced	H3K9	PASMCs	Increases proliferation due to excessive activation of cell cycle progression	[30]
MEG3	↑	Mice, Humans	Hypoxia -induced	miR-328-3p/IGF1R	PASMCs	Excessive activation of cell cycle progression and promotion of proliferation.	[31]
TYKRIL	↑	Humans	PCLS	p53/PDGFRβ	hPASMCs	Promotes proliferation and inhibits apoptosis	[32]
SOX2-OT	↑	Humans	Cell culture	–	hPASMCs	Promotes proliferation, migration, apoptosis resistance, and inflammatory responses	[33]
LincRNA- COX2	↑	Mice	Hypoxia -induced	miR-let- 7a/STAT3	PASMCs	Promotes proliferation	[34]
SMILR	↑	Humans	Cell culture, Hypoxia -induced	miR-141/RhoA/ROCK	hPASMCs	Promote cell proliferation-related signaling	[35]
AC068039.4	↑	Humans	Cell culture	miR-26a-5p/TRPC6	hPASMCs	Promotes proliferation and cell cycle progression	[36]
LINC00963	↑	Mice	Hypoxia -induced	miR-328-3p/PFN1	PASMCs	Promotes cell viability and enhances migration	[37]
NONRATT015587.2	↑	Rats	MCT-induced	p53/HIF-1	PASMCs	Increases the proportion of cells in the S and G2/M phases to promote proliferation	[38]
NEAT1	↑	Humans	Cell culture, Hypoxia -induced	miR-34a-5p/KLF4	hPASMCs	Promotes proliferation and migration	[39]
PVT1	↑	Rats	Hypoxia -induced	miR-186/Srf/Ctgf and miR-26b/Ctgf	PASMCs	Regulates autophagy and increases cell proliferation	[40]
UCA1	↑	Rats	Hypoxia -induced	ING5/hnRNP I	PASMCs	Promotes proliferation and inhibits apoptosis	[41]

MALAT1, metastasis-associated lungadenocarcinoma transcript 1; Lnc-Ang362, lncRNA 362 regulated by angiotensin II; PAXIP1-AS1, lncRNA 362 regulated by angiotensin II; TUG1, taurine-upregulated gene 1; HOXA-AS3, lncRNA cluster antisense RNA 3; MEG3, maternally expressed gene 3; TYKRIL, tyrosine kinase receptor inducing lncRNA; SOX2-OT, lncRNA SOX2-overlapping transcript; LincRNA- COX2, long intergenic non-coding RNA COX2; SMILR, smooth muscle enriched long noncoding RNA; LINC00963, long intergenic non-protein coding RNA 963; NEAT1, nuclear paraspeckle sssembly transcript 1; PVT1, plasmocytoma variant translocation 1; UCA1, urothelial carcinoma associated 1; MCT, monocrotaline; hPASMCs, human pulmonary artery smooth muscle cells; PDGF-BB, platelet-derived growth factor-BB; AT1R, Ang II type 1 receptor; KLF5, Kruppel-like factor 5; NF-kB, nuclear factor-kappaB; TLR4, Toll-like receptor 4; ETS1, E26 transformation specific1; WIPF1, Wiskott-Aldrich syndrome protein interacting protein family member 1; RhoA, Ras homolog gene family member A; Foxc1, Forkhead box C1; H3K9, histone H3 lysine 9; IGF1R, insulin-like growth factor 1 receptor; PCLS, precision-cut lung slices; p53, tumor protein 53; PDGFRβ, platelet-derived growth factor receptor β; TRPC6, transient receptor potential canonical 6; PFN1, profilin 1; HIF-1, hypoxia-inducible factor-1 ; KLF4, Kruppel-like factor 4; Srf, serum response factor ; Ctgf, Connective tissue growth factor; ING5, inhibitor of growth 5; hnRNP I, heterogeneous nuclear ribonucleoprotein 1; STAT3, signal transducer and activator of transcription 3; ROCK, Rho-associated protein kinase; PASMCs, pulmonary artery smooth muscle cells.

**Table 2.** **LncRNAs that inhibit the proliferation and migration of PASMCs**.

LncRNA	Regulation	Species	Models established	Targets	Cells	Functions	Ref
LnRPT	↓	Rats	MCT-induced	PDGF-PI3K-LnRPT-Notch3	PASMCs	Inhibits proliferation	[42]
CASC2	↓	Mice	Hypoxia -induced	α-SMA	PASMCs	Inhibits proliferation, migration and vascular remodeling	[43]
		Humans	Cell culture, Hypoxia -induced	miR-222/ING5	hPASMCs	Inhibits proliferation and migration and prevents vascular remodeling	[44]
Rps4l	↓	Mice, Humans	Cell culture, Hypoxia -induced	RPS4XL/RPS6	PASMCs	Inhibita proliferation	[45]
		Mice	Hypoxia -induced	ILF3/HIF-1α		Regulates proliferation, migration, and cell cycle progression	[46]
		Mice		HSC70		Inhibits hypoxia-induced pyroptosis	[47]
MEG3	↓	Humans	Hypoxia -induced	p53	hPASMCs	Inhibits proliferation and migration	[48]
			Cell culture, Hypoxia -induced	miR-21/PTEN			[49]
Gas5	↓	Rats	Hypoxia -induced	miR-23b-3p/KCNK3	PASMCs	Inhibits proliferation and migration	[50]
		Humans	PDGF-BB treatment	miR-382-3p	hPASMCs	Mediates the regulation of pulmonary artery remodeling and autophagy in PASMCs in CTEPH	[51]
CPS1-IT	↓	Rats	OSA	HIF-1/NF-kB/IL-1β	PASMCs	Inhibits proliferation	[52]
ANRIL	↓	Humans	Hypoxia -induced	–	hPASMCs	Inhibits proliferation and migration	[53]
TCONS_00034812	↓	Rats	Hypoxia -induced	Stox1/MAPK	PASMCs	Inhibits proliferation and promotes apoptosis	[54]
PAHRF	↓	Humans	Hypoxia -induced	miR-23a-3p/MST1	hPASMCs	Inhibits proliferation and promotes apoptosis	[55]
LncPTSR	↓	Rats	Cell culture	PDGF-BB/MEK/ERK/PMCA4/${\mathrm{Ca}}^{2+}$	PASMCs	Participates in pulmonary artery remodeling as an important regulator of PDGF and calcium signaling	[56]

LnRPT, lncRNA regulated by PDGF and transforming growth factor β; CASC2, cancer susceptibility candidate gene 2; Rps4l, ribosomal protein S4-like; MEG3, maternally expressed gene 3; Gas5, growth arrest-specific 5; CPS1-IT1, CPS1 intron transcript 1; ANRIL, antisense noncoding RNA in the INK4 locus; PAHRF, pulmonary arterial hypertension related factor; LncPTSR, lncRNA neighboring the locus of ATPase plasma membrane Ca2+ transporting 4; MCT, monocrotaline; hPASMCs, human pulmonary artery smooth muscle cells; PDGF, platelet derived growth factors; PDGF-BB, platelet-derived growth factor-BB; PI3K, phosphatidylinositol 3-kinases; α-SMA, alpha-smooth muscle actin; ING5, inhibitor of growth 5; RPS4XL, peptide 40S ribosomal protein S4 X isoform-like; RPS6, ribosomal protein S6; ILF3, interleukin enhancer-binding factor 3; HSC70, heat-shock cognate protein 70; p53, tumor protein 53; PTEN, phosphatase and tensin homolog; KCNK3, potassium channel subfamily K member 3; CTEPH, chronic thromboembolic pulmonary hypertension; OSA, obstructive sleep apnea; HIF-1, hypoxia-inducible factor-1; NF-kB, nuclear factor-kappaB; IL-1β, interleukin-1beta; Stox1, storkhead box 1; MAPK, mitogen-activated protein kinases; MST1, Mammalian sterile 20-like kinase 1; MEK, mitogen-activated protein kinase; ERK, extracellular regulated kinase; PMCA4, plasma membrane calcium/calmodulin dependent ATPase isoform 4.

#### 3.1.1 LncRNAs that Promote PASMC Proliferation and Migration

3.1.1.1 LncRNA H19

H19 is one of the earliest identified imprinted lncRNAs and is thought to be an embryonic or a tumor suppressor gene [57]. H19 is upregulated in the rat lung after monocrotaline (MCT) treatment. Upregulation of H19 was associated with PDGF-BB, and after platelet-derived growth factor type-BB (PDGF-BB) stimulation, H19 interacted with the miRNA let-7b to increase angiotensin II receptor type 1 (AT1R) expression levels by sponging let-7b; in addition, AT1R is a new target of let-7b, and previous reports have shown that AT1R is important for increasing vascular proliferation through activation of mitogen-activated protein kinases (MAPK) and RhoA signaling [58]. Moreover, let-7b is involved in inhibiting PASMC proliferation in hypoxic pulmonary hypertension (HPH) [58]. A study by Su *et al*. [23] confirmed that the H19-let-7b-AT1R axis participates in the pathogenesis of PH by stimulating PASMC proliferation.

3.1.1.2 MALAT1: LncRNA Metastasis-Associated Lung Adenocarcinoma Transcript 1

In 2017, Brock *et al*. [59] demonstrated that MALAT1 was upregulated in hypoxic human PAECs (hPAECs) and human PASMCs (hPASMCs) as well as in the lungs of hypoxic mice and reported that MALAT1 expression is driven by hypoxia-inducible factor 1a (HIF1a), that PASMC proliferation is regulated by the expression of cell cycle protein-dependent kinase (CDK) inhibitors and that MALAT1 controls the PASMC phenotype mainly by targeting CDK-related phenotypic characteristics; however, the specific mechanism by which MALAT1 regulates vascular smooth muscle cell proliferation was not clarified in that study. Subsequently, Wang *et al*. [24] reported upregulated MALAT1 expression in PASMCs isolated from PH patients, and in that study, MALAT1 was found to promote the proliferation of PASMCs by sponging miR-124-3p.1 and upregulating the expression of the downstream target gene Kruppel-like factor 5 (KLF5), indicating that MALAT1 promotes pulmonary vascular remodeling and cell cycle progression in PH through the miR-124-3p.1/KLF5 axis. Moreover, MALAT1 participates in the pathological processes of PH through other regulatory signaling axes or signaling networks. Recent studies have suggested that MALAT1 promotes the proliferation and migration of hPASMCs by modulating the miR-503/Toll-like receptor 4 (TLR 4) signaling axis. Mechanistically, MALAT1 expression is high in the plasma of PH patients and in hypoxic hPASMCs, while miR-503 expression is low. MALAT1 acts as a ceRNA for miR-503, regulates the downstream target gene of miR-503, and TLR4, promotes the proliferation and migration of hPASMCs, and inhibits their apoptosis. These findings suggest that MALAT1 is involved in PH pathogenesis by inhibiting miR-503/TLR 4 signaling axis [25].

3.1.1.3 Lnc-Ang362: LncRNA 362 Regulated by Angiotensin II

Wang *et al*. [26] reported the overexpression of lnc-Ang362, miR-221, and miR-222 in lung tissues and hypoxic hPASMCs from PH patients. Upregulation of lnc-Ang362 enhanced hPASMC proliferation and migration. Mechanistically, lnc-Ang362 upregulated the expression of miR-222 and miR-221 in hPASMCs and in turn regulated the proliferation and migration of hPASMCs by activating the NF-κB signaling pathway. Therefore, lnc-Ang362 could be a new lncRNA candidate for the treatment of PH.

3.1.1.4 PAXIP1-AS1: LncRNA PAXIP1 Antisense RNA 1

PAXIP1-AS1 plays an intrinsic role in coordinating the hyperproliferation and migratory activity of smooth muscle cells in patients with idiopathic pulmonary arterial hypertension (iPAH). PAXIP1-AS1 acts on the downstream target paxillin and disrupts the focal adhesion axis by regulating its expression and phosphorylation, leading to increased PASMC proliferation [27]. A recent study revealed that PAXIP1-AS1 also acts on PASMCs through another signaling axis. Specifically, PAXIP1-AS1 and RhoA were significantly overexpressed in the lung tissues and serum of rats with MCT-induced PH and in hypoxic hPASMCs. Mechanistically, PAXIP1-AS1 forward regulates WIPF1 by aggregating the transcription factor ETS1, and PAXIP1-AS1-mediated biological functions can be reversed by knocking down E26 transformation specific 1 (ETS1). The mutual effect of WIPF1 and RhoA was verified by coimmunoprecipitation. In conclusion, PAXIP1-AS1 promotes the proliferation and migration of hPASMCs through the ETS1/WIPF1/RhoA axis, participating in PH pathogenesis [28].

3.1.1.5 LncRNA Taurine-Upregulated Gene 1 (LncRNA TUG1)

TUG1, a recently identified oncogenic lncRNA with a length of approximately 7.1 kb [60], has been found to be abnormally upregulated in different types of cancer [61]. Lv *et al*. [62] showed that aberrant expression of TUG1 may be involved in the regulation of pulmonary vascular cell function under hypoxic conditions. During the pathological development of PH, TUG1 targets multiple miRNAs, suggesting that it may exert combinatorial effects on multiple factors and signaling pathways to cause occlusion of small PAs. TUG1 was highly expressed in mice with HPH and in PSMCs from these mice (HPH-PASMCs), and silencing TUG1 inhibited the proliferation and migration of PASMCs and promoted their apoptosis. TUG1 can bind to miR-374c and upregulate downstream Foxc1 expression, and silencing lncRNA TUG1 attenuates pulmonary vascular remodeling in mice with HPH through Foxc 1-mediated NOTCH signaling. Taken together, these results suggest that the lncRNA TUG1 participates in pulmonary vascular remodeling by binding to miR-374c to control Foxc1 and Notch signaling to in turn regulate PASMC proliferation, migration, and apoptosis [29]. In addition, Bonnet *et al*. [63] reported that TUG1 was upregulated in the lung tissues of PH patients and mice with chronic hypoxia exposure, in which TUG1 acted as a ceRNA to mediate PASMC proliferation by sponging miR-328-3p. In addition, TUG1 has been reported to act as a molecular sponge for other miRNAs, including miR-223, 10 miR-29, and miR-204.

3.1.1.6 LncRNA Cluster Antisense RNA 3 (HOXA-AS3)

HOXA-AS3 was found to be overexpressed in the pulmonary vessels and PASMCs of mice with hypoxia- and MCT-induced PH. Acetylation of histone H3 lysine 9 (H3K9), located in the promoter region, promotes the transcription of HOXA-AS3. High expression of HOXA-AS3, which controls the cell cycle by upregulating Hoxa 3 at the transcriptional and translational levels, is linked to cell proliferation, and thereby promotes PASMC proliferation [64]. HOXA-AS3 was also found to regulate PASMC proliferation and migration via other signaling axes. In the presence of HYP, HOXA-AS3 and PDE5A were upregulated while miR-675-3p expression, on the other hand, was decreased. Furthermore, after overexpressing miR-675-3p, knockdown of HOXA-AS3 inhibited HPASMC development and migration but caused apoptosis miR-675-3p. Inhibiting miR-675-3p or increasing PDE5A expression effectively reversed the inhibitory effect of HOXA-AS3 knockdown on PH. Mechanistically, HOXA-AS3 directly promotes PASMC proliferation by targeting PDE5A through sponging of miR-675-3p. In conclusion, HOXA-AS3 promotes the occurrence of PH by regulating the miR-675-3p/PDE 5 axis and may be a therapeutic biomarker in PH [30].

3.1.1.7 Long Noncoding RNA-Maternally Expressed Gene 3 (MEG3)

MEG3 is an oncogenic 1.6 kb lncRNA and is a key regulator of PH [65]. In iPAH patients and associated HPH models, MEG3 and IGF1R are overexpressed, while miR-328-3p is not expressed at high levels. Mechanistically, MEG3 was found to interact with and lead to the degradation of miRNA-328-3p, contributing to the upregulation of insulin-like growth factor 1 receptor (IGF1R), which promotes PASMC proliferation and cell cycle progression [31]. Notably, in a 2019 study by Xing *et al*. [31] revealing the pathogenic relevance of the lncRNA MEG3 in PH, with completely opposite results to a 2017 study [66]. To explain these inconsistent results, Xing *et al*. [31] proposed numerous hypotheses: First, the reason may be related to the different selections of the 15 MEG3 transcript variants. Second, MEG3 expression was upregulated in hypoxia-stimulated PASMCs but downregulated in other tissues as well as in lung cells, suggesting that this lncRNA may exhibit tissue- or cell-specific expression. The above two items may have contributed to the inconsistent results of these studies [31, 66].

3.1.1.8 Tyrosine Kinase Receptor Inducing LncRNA (TYKRIL)

Using RNAseq data, Zehendner *et al*. [32] discovered a new lncRNA, TYKRIL, that was significantly upregulated in all four hyperproliferative situations in PASMCs and pericytes from iPAH patients as well as in PASMCs and pericytes exposed to hypoxia. TYKRIL promotes proliferation and inhibits apoptosis by binding to the tumor suppressor p53 and by promoting platelet-derived growth factor receptor (PDGFR) transcription, thereby promoting proliferation and inhibiting apoptosis in PASMCs and pericytes.

3.1.1.9 LncRNA SOX2-Overlapping Transcript (SOX2-OT)

The serum SOX2-OT concentration was found to be high in PH patients, and elevated levels of SOX2-OT levels had a significant ability to distinguish PH patients from healthy controls, suggesting that SOX2-OT may be a viable diagnostic marker for PH. In hypoxic hPASMCs, it was discovered that SOX2-OT expression increased in a time-dependent manner. In addition, SOX2-OT knockdown reversed the effects of hypoxia on the inhibition of hPASMC apoptosis, proliferation, migration, and inflammatory responses. The exact mechanism is not clear at this time. However, the results of rescue experiments showed that the reversal of these effects by SOR2-OT silencing was attenuated by the inhibition of miR-455-3p, an effect possibly mediated through small ubiquitin-like modifier 1 (SUMO1) [33].

3.1.1.10 Long Intergenic Noncoding RNA COX2 (LincRNA-COX2)

LincRNA-COX2 was found to be upregulated in peripheral blood-like and hypoxic PASMCs from PH patients. Silencing of lincRNA-COX2 inhibited hypoxia-induced proliferation of PASMCs by affecting progression through the G2/M transition through the cell cycle. Mechanistically, by acting on the downstream target STAT3 through miR-let-7a, lincRNA-COX2 influences the phenotype of PASMCs. As a result, its impact on hypoxic PASMCs is mediated by the miR-let-7a/STAT3 axis [34].

3.1.1.11 Smooth Muscle Enriched Long Noncoding RNA (SMILR)

The expression of the lncRNA SMILR was found to be increased in PH patients and in *in vivo* and *in vitro* models. In a study by Lei *et al*. [35], the expression of SMILR and RhoA was increased but miR-141 expression was downregulated in a rat model of MCT-induced PH. SMILR could directly negatively regulates miR-141 expression, and when SMILR is silenced, miR-141 is overexpressed and inhibits the RhoA/ROCK pathway by binding to RhoA, thereby suppressing cell proliferation-related signaling. In conclusion, the lncRNA-SMILR modulates the RhoA/ROCK signaling pathway by targeting miR-141 to regulate vascular remodeling in PAH [35].

3.1.1.12 LncRNA AC068039.4

Overexpression of the lncRNA AC068039.4 contributes to PASMC proliferation and cell cycle progression by sponging miR-26a-5p/TRPC 6 in the context of HPH. Verification by qPCR confirmed that AC068039.4 was significantly upregulated in hypoxia-induced PASMCs and that miR-26a was downregulated in the plasma of PH patients and in the lung tissues of rats with MCT-induced PH. Knockdown of AC068039.4 attenuates PASMC proliferation and migration and regulates cell cycle progression by inhibiting G0- and G1-phase entry. Furthermore, further experiments showed that AC068039.4 interacts with and sponges miR-26a-5p, resulting in reduced TRPC6 degradation and thus reducing the involvement of TRPC6 in the conversion of PASMCs from a contractile to a proliferative phenotype [36, 67]. Upregulation of TRPC6 is associated with PASMC proliferation and pulmonary vascular resistance (PVR), and increased expression of TRPC6 ultimately leads to PASMC hyperproliferation [68]. These findings suggest a new therapeutic approach for HPH.

3.1.1.13 Long Intergenic Non-Protein Coding RNA 963 (LINC00963)

In hypoxia-exposed PASMCs and mouse models of PH, the levels of LINC00963 and profilin 1 (PFN1) were found to be elevated, while the miR-328-3p level was decreased. LINC00963 acts as miR-328-3p sponge, whereas PFN1, a downstream target of miR-328-3p, is a ubiquitously expressed actin-binding protein that regulates cell differentiation, proliferation and motility [69]. Overexpression of PFN1 is closely associated with exacerbation of PH. LINC00963 silencing decreased the viability and inhibited the migration of PASMCs. These results indicate that LINC00963 is involved in the progression of PAH by regulating the miR-328-3p/PFN1 axis [37].

3.1.1.14 LncRNA NONRATT015587.2

The analysis showed that NONRATT015587.2 plays a role in pulmonary vascular remodeling, and the overexpression of NONRATT015587.2 *in vitro* promoted the proliferation of PASMCs and upregulated the ratio of S-phase to G2/M-phase cells, whereas the knockdown of NONRATT015587.2 promoted the apoptosis of PASMCs by disrupting the integrity of mitochondria, and both of which are associated with vascular remodeling in PH. Moreover, the p53 and HIF-1 signaling pathways were involved in NORNATT015587.2-induced vascular remodeling. It is possible that the antidiabetic drug metformin exerts its therapeutic effect on hypoxia-induced PH in mouse and rat models by modulating the expression of the lncRNA NONRATT015587.2 [38].

3.1.1.15 Nuclear Paraspeckle Assembly Transcript 1 (NEAT1)

Dou *et al*. [39] examined the expression levels of NEAT1, KLF4 and miR-34a-5p in the serum of hypoxia-treated PASMCs and PH patients and reported increased expression of NEAT1 and KLF4 but decreased expression of miR-34a-5p. NEAT1 targets miR-34a-5p, whereas miR-34a-5p targets KLF 4. Transfection of sh-NEAT1 or miR-34a-5p mimics reduced the proliferation and migration of hypoxia-treated PASMCs. The inhibitory effect of NEAT1 knockdown on the proliferation and migration of hypoxia-treated PASMCs was reversed by downregulation of miR-34a-5p expression and increased KLF4 expression. These findings suggested that the NEAT 1/miR-34a-5p/KLF 4 axis is involved in PH pathogenesis.

3.1.1.16 Long Noncoding RNA Plasmocytoma Variant Translocation 1 (LncRNA PVT1)

The lncRNA PVT1 is an oncogene that is highly expressed in a variety of cancers including gastric cancer, human glioma, and non-small cell lung cancer [70]. Recent research has shown that hypoxia-induced upregulation of the lncRNA PVT1 regulates autophagy through the miR-186/Srf/Ctfgf and miR-26b/Ctgf signaling pathways, exacerbating PASMC proliferation. It has been reported that the lncRNA PVT1 acts as a ceRNA for miR-186 and miR-26b in different tissue types and that miR-186 and miR-26b are dysregulated in hypoxia-induced PASMCs and involved in the pathogenesis of HPH. Xia *et al*. [71] observed that hypoxia significantly altered the expression of PVT1, serum response factor (SRF), connective tissue growth factor (CTGF), miR-26b and miR-186 in a rat model. Luciferase assays confirmed that Srf mRNA and PVT1 may interact with miR-186 and that miR-26b may interact with PVT1 and CTGF mRNA. Moreover, the upregulation of PVT1 reduced the levels of miR-186 and miR-26b but increased the expression of LC3B-II, CTGF, and SRF. SRF is a representative transcription factor that plays an important role in the angiogenesis process [40]. CTGF is a virtual target gene of miR-26b, and miR-26b attenuates monoclonal-induced pulmonary vascular remodeling by targeting CTGFs and cyclin D1 (CCND 1). In the HPH rat model, hypoxia induced miR-26b inhibition and upregulation of SRF and CTGF. CTGF mediates the regulation of PASMC proliferation by miR-26b and SRF, suggesting that hypoxia-induced inhibition of miR-26b contributes to the pathogenesis of HPH through CTGF. In conclusion, these results showed that in the HPH model, PVT1 may be involved in hypoxia-induced proliferation of PASMCs by regulating the miR-186/Srf/Ctgf and miR-26b/Ctgf signaling pathways [71].

3.1.1.17 Urothelial Carcinoma Associated 1 (UCA1)

UCA1 is an oncogenic lncRNA that was first identified in bladder cancer and is highly expressed in a variety of cancers, including gastric cancer, colorectal cancer, lung cancer and breast cancer [72]. Moreover, studies suggest a potential role for UCA 1 in the treatment of HPH. Zhu *et al*. [41] simulated PH *in vitro* and reported that UCA1 was highly expressed under hypoxic conditions, promoted the proliferation of HPASMCs and inhibited their apoptosis. Further mechanistic studies revealed that UCA1 competes with ING5 for binding to hnRNP I and that ING5 inhibits cell viability but promots apoptosis; thus, upregulation of UCA1 inhibited the protein expression of ING5. In conclusion, UCA1 promotes cell proliferation and suppresses apoptosis by competing with ING5 for binding to hnRNP I in hypoxia-induced HPASMCs, suggesting a potential role for UCA1 in the treatment of HPH.

#### 3.1.2 LncRNAs that Inhibit PASMC Proliferation and Migration

3.1.2.1 LnRPT: LncRNA Regulated by PDGF and Transforming Growth Factor β (TGF-β)

After RNA-seq analysis, qRT‒PCR analysis and other functional validation, Chen *et al*. [73, 74] selected an antiproliferative lncRNA downregulated by PDGF and TGF-β stimulation in rat PASMCs and named it lnRPT. In addition LnRPT was found to be downregulated in pulmonary arteries in the context of MCT-induced PH; consistent these findings, LnRPT was also downregulated in hPASMCs after 12 h of PDGF-BB treatment, indicating that LnRPT is regulated by PDGF and downregulated by PDGF-BB through the PI3K pathway [42]. LnRPT strongly inhibited effect on PASMC proliferation. Mechanistically, LnRPT suppresses the expression of two genes, notch3, Notch ligand 1 (jag 1), and the cell cycle regulator ccna 2, which regulate cell cycle progression to inhibit PASMC proliferation and play a role in the development of PH [74]. These results suggest a role for the PDGF-PI3K-LnRPT-Notch3 signaling axis in the pathobiology of PH.

3.1.2.2 LncRNA Cancer Susceptibility Candidate Gene 2 (LncRNA CASC2)

CASC2 expression was decreased in hypoxia-exposed rat pulmonary artery tissues and PASMCs. Upregulation of CASC2 inhibited cell proliferation and migration but enhanced apoptosis in the context of hypoxia-induced PH *in vitro* and *in vivo*. Gong *et al*. [43] showed that upregulation of the lncRNA CASC2 in the context of hypoxia-induced PH markedly reduced the expression of the phenotypic switch switch α-SMA. Furthermore, it was shown by pulmonary artery morphometric analysis revealed that the lncRNA CASC2 inhibited hypoxia-induced vascular remodeling in rat pulmonary artery tissue. In a new study by Han *et al*. [44], an alternative signaling axis by which CASC2 regulates the PASMC phenotype was identified, and CASC2 was observed to be downregulated in hypoxia-exposed PASMCs in a dose- and time-dependent manner. Mechanistically, CASC2 can act as a ceRNA for miR-222, thus adjusting the expression level of ING5, a downstream target of miR-222, in PASMCs. Furthermore, the results of rescue assays showed that the inhibitory effect of CASC2 on the hypoxia-induced proliferation and migration of PASMCs was attenuated by the inhibition of miR-222 or upregulation of ING5. In conclusion, CASC2 can inhibit hypoxia-induced proliferation and migration of PASMCs by regulating the MIR-222/ING5 axis, and impeding the progression of PH, these findings provide new insights and therapeutic strategies for hypoxia-induced PH.

3.1.2.3 LncRNA Ribosomal Protein S4-Like (Rps4l)

Rps4l expression was found to be considerably lower in hypoxic PASMCs and PH model mice. Liu *et al*. [45] established transgenic mice overexpressing Rps4l (Rps4lTg mice) and reported that, at the cellular level, the overexpression of Rps4l decreased cell viability and proliferation and impeded cell cycle progression. The results of *in vitro* experiments showed that the expression of Rps4l was negatively correlated with that of interleukin-enhancing binding factor 3 (ILF3) and that the overexpression of Rps4l enhanced ILF3 degradation and affected Hif1a mRNA expression, leading to the inhibition of PASMC proliferation and migration. In summary, in hypoxic PASMCs, Rps4l expression was reduced due to the regulatory effects of hypoxia, and this reduction affected PASMC proliferation, migration and cell cycle progression via ILF3/HIF-1α. Subsequent studies showed that RPS4L has protein-coding ability, acting not only alone but also through its encoded peptide. Li *et al*. [46] demonstrated that lnc-Rps4l has an ORF with the protein-coding capacity of Rps4l that encodes the 40S ribosomal protein S4 X isomer (RPS4XL) peptide, which can regulate proliferation and pyroptosis through different signaling pathways, leading to pulmonary vascular remodeling. RPS4XL expression is reduced in hypoxia-induced PH and hypoxic PASMCs, and phosphorylation of RPS 6 is a key event in the promotion of PASMC proliferation and migration; RPS4XL suppresses hypoxia-induced PASMC proliferation by inhibiting the binding of RPS6 after its phosphorylation, suggesting that the novel RPS4XL peptide plays a crucial role in regulating hypoxia-induced PASMC proliferation. In addition, RPS4XL is involved in the degradation process in PH. Pyroptosis is one of the modes of programmed cell death and manifests as continuous swelling of the cell until the cell membrane ruptures, leading to the release of cellular contents and the activation of a robust inflammatory response [75]. Recent studies have shown that pyroptosis occurs in the pulmonary arterial media in a PH rat model and in hypoxic hPASMCs [76]. LncRNAs play important roles in regulating pyroptosis and are involved in this process by directly or indirectly acting on proteins associated with the pyroptosis signaling pathway [77, 78, 79]. Li *et al*. [47] demonstrated the role of the RPS4XL peptide encoded by lnc-Rps4l and its regulatory mechanism in pyroptosis in the context of PH, this peptide was induced in transgenic mice overexpressing lnc-Rps4l to reverse the inhibition of hypoxia-induced pyroptosis in PH mouse models and hypoxic PASMCs. A study showed that in transgenic mice, overexpression of lnc-Rps4L restored the inhibition of hypoxia-induced pyroptosis in PASMCs and experimentally confirmed that RPS4XL inhibits pyroptosis in a PH mouse model and in hypoxic PASMCs by regulating HSC70 glycosylation. RPS4XL can inhibit hypoxia-induced proliferation of PASMCs but does not oppose RPS4XL-mediated inhibition of hypoxia-induced pyroptosis in PASMCs. In hypoxia, PASMC proliferation causes vascular wall thickening, and pyroptosis induces increased fibrosis, leading to PVR [47].

3.1.2.4 Maternally Expressed Gene 3 (MEG3)

MEG3 was also shown to be downregulated in the lungs of PH patients and in PAs, and MEG3 deletion regulated cell cycle progression, allowing more smooth muscle cells to complete the G0/G1 transition and enter G2/M+S phase and accelerating the proliferation of PASMCs, thus stimulating the expression of PCNA, Cyclin A, and Cyclin E. The MEG3 pathway was found to be involved in the proliferation of PASMCs. In addition, the p53 pathway was found to be involved in MEG3-induced smooth muscle cell proliferation [48]. Further studies showed that MEG3 exerts its effects under both normoxic and hypoxic conditions by regulating the expression of miR-21, which regulates PTEN, and the final results showed that MEG3 plays a role in human PASMCs under both normoxic and hypoxic conditions through the miR-21/PTEN axis [49].

3.1.2.5 Growth Arrest-Specific 5 (Gas5)

Gas5 was downregulated in a hypoxic rat model and in cultured hypoxic hPASMCs. Silencing Gas5 significantly promoted hPASMC proliferation and migration under both normoxic and hypoxic conditions. Mechanistically, miR-23b-3p interacts directly with miRNA binding sites in the Gas5 sequence, and Gas5 functions as a ceRNA for miR-23b-3p to regulate KCNK3 expression; these interactions promote hPASMC proliferation and migration [50]. In addition, cellular models were constructed by Feng *et al*. [51] by treating PASMCs with PDGF-BB, and GAS5 was found to promote autophagy through the inhibition of PAMSC functions, mean pulmonary arterial pressure (mPAP), pulmonary artery wall thickening and angiogenesis in rats with chronic thromboembolic pulmonary hypertension (CTEPH) by targeting downstream miR-382-3p. These findings show that the GAS 5/miR-382-3p axis is involved in the regulation of pulmonary artery remodeling and autophagy in CTEPH.

3.1.2.6 LncRNA CPS1 Intron Transcript 1 (CPS1-IT)

The lncRNA CPS1-IT is a novel tumor suppressor [80]. In addition, CPS1-IT was found to be downregulated and IL-1β was found to be upregulated in the pulmonary artery tissue of obstructive sleep apnea (OSA) model rats. Overexpression of CPS1-IT reduces IL-1β expression by inhibiting the transcriptional activity of HIF1, thereby inhibiting the NF-κB signaling pathway. Mechanistically, HIF-1 is a heterodimeric protein composed of two subunits, HIF-1α and HIF-1β, and HIF-1α is expressed in smooth muscle cells and plays a key role in hypoxia-induced PH in mice. Previous studies have suggested that inhibition of HIF-1α accumulation during inflammation can lead to blockade of IL-1β induction, with IL-1β increasing HIF-1α protein expression under normoxic conditions and activation of HIF-1α-mediated vascular endothelial growth factor expression induced via the NF-κB-dependent pathway [81]. In rats with MCT-induced PH, NF-κB-mediated autophagy was significantly activated, and inhibition of NF-κB activation attenuated both autophagy and vascular remodeling [82]. Thus, it was concluded that the CPS1-IT/HIF-1/IL-1β axis affects PH in rat OSA model via the NF-κB signaling pathway [52].

3.1.2.7 Antisense Noncoding RNA in the INK4 Locus (ANRIL)

ANRIL is a key regulator of hypoxia-exposed HPASMCs and is significantly downregulated in hypoxia-exposed HPASMCs. Downregulation of ANRIL affected the cell cycle, allowing more hPASMCs to progress through the G0/G1 transition to the G2/M+S phase and increasing cell proliferation. Furthermore, downregulation of ANRIL increased HPASMC migration under hypoxic conditions [53].

3.1.2.8 TCONS_00034812

Real-time fluorescence quantitative PCR showed that TCONS_00034812 was significantly downregulated in pulmonary arteries of PH rats and in hypoxic PASMCs. TCONS_00034812 upregulated the expression of the transcription factor Stox1, and silencing TCONS_00034812 promoted the proliferation and inhibited the apoptosis of PASMCs *in vitro*. Knockdown of TCONS_00034812 and Stox1 regulated PASMC functions through MAPK signaling. These results suggest that lncRNA-TCONS_00034812 participates in vascular remodeling during PH and regulates the proliferation and apoptosis of PASMCs through the Stox1/MAPK signaling pathway [54].

3.1.2.9 LncRNA Pulmonary Arterial Hypertension Related Factor (PAHRF)

Both PH patients and HPASMCs subjected to hypoxia were shown to express PAHRF. PAHRF interacts with miR-23a-3p; is involved in the regulation of hypoxia-induced hPASMC proliferation, and apoptosis, and in cell cycle progression; and is a key regulator of HPH. Mechanistic studies have shown that PAHRF acts as a sponge of miR-23a-3p, which targets MST1, and that PAHRF thus suppresses MST1 expression by competitively binding to miR-23a-3p, promoting PASMC apoptosis and inhibiting PASMC proliferation; these findings suggest that PAHRF participates in pulmonary vascular remodeling in HPH through the miR-23a-3p-MST1 signaling axis [55].

3.1.2.10 LncRNA Neighboring the Locus of ATPase Plasma Membrane Ca2+ Transporting 4 (LncPTSR)

Vascular remodeling and vasoconstriction in PH are associated with elevated intracellular calcium ion concentrations, and PDGF-BB is the most potent mitogen in PASMCs and is involved in vascular remodeling. PDGF signaling has been shown to be involved in maintaining Ca2+ homeostasis in PASMCs [83]. lncPTSR is a highly conserved nuclear lncRNA that is downregulated in PASMCs in response to PDGF-BB stimulation. lncPTSR negatively regulates the proliferation, apoptosis, and migration of rat PASMCs, and its knockdown inhibits the expression of plasma membrane Ca2+ transporting 4 (PMCA4) and attenuates Ca2+ efflux from PASMCs *in vitro* and *in vivo*. Mechanistic studies demonstrated a complex interaction between lncPTSR and the mitogen-activated protein kinase (MEK) pathway: inhibition of mitogen-activated protein kinase kinase and extracellular signal-regulated kinase (MEK/ERK) suppressed PDGF-BB-mediated downregulation of lncPTSR, and lncPTSR acted as a feedback regulator of MEK signaling molecules. When lncPTSR was downregulated, PMCA4 expression was no longer increased by nuclear lncPTSR, resulting in decreased PMCA4 levels and increased intracellular Ca2+ levels. An imbalance in intracellular calcium levels increases vascular tone and drives cells in the vasculature toward a proliferative and antiapoptotic phenotype. Taken together, these results indicate that lncPTSR is involved in pulmonary artery remodeling by regulating PMCA4 expression and intracellular Ca2+ homeostasis downstream of PDGF-BB-driven MEK/ERK signaling [56].

### 3.2 LncRNAs and EC in PH

PAECs are involved in the development and progression of PH by promoting lung inflammation and coagulation, oxidative stress, proliferation, metabolic dyshomeostasis, and the accumulation of inflammatory cells and fibroblasts [84, 85]. Many features of PH are consequences of dysfunctional EC signaling [86]. The early stages of PH development involve EC damage and apoptosis, and as the disease progresses, apoptosis-resistant endothelial cells (ECs) develop [87, 88]. Finally, in the advanced stages of PH, EC hyperproliferation and apoptosis resistance are the dominant processes involved in PH and contribute to the formation of plexiform lesions [89, 90]. Recent have indicated that several lncRNAs are involved in vascular remodeling in PH by regulating PAEC proliferation, migration, apoptosis, autophagy, pyroptosis and EndMT (Table 3, Ref. [91, 92, 93, 94, 95, 96, 97, 98]).

**Table 3.** **LncRNAs acting on ECs**.

LncRNA	Regulation	Species	Models established	Targets	Cells	Functions	Ref
Xist	↑	Mice	${\mathrm{EH}}_{\text{ITSN}}$-KO^ITSN+/-^ mouse model of plexiform arteriopathy	p38-ELK1-c-Fos	PAECs	Involvement in the endothelial cell proliferative response sexually dimorphic	[97, 98]
MIAT	↑	Rats	MCT induced	miR29a-5p/Nrf2	PAECs	Promotes proliferation and migration and aggravates oxidative stress in the HPH model	[91]
GAS5	↑	Humans	Cell culture	miRNA-31-5p/NAT8L	PAECs	Promotes autophagy induced by SP in PAEC	[92]
AERRIE	↑	Humans	Cell culture	–	HUVECs	Induces EndMT, regulating mesenchymal markers and transcription factors	[93]
MALAT1	↑	Humans	Cell culture	MALAT1-miR-145-TGFBR2/Smad3	EPC	Regulates TGF-β1-induced EndMT	[94]
			TGF-β1 treatment				
GATA6-AS	↑	Humans	Cell culture, Hypoxia -induced	LOXL2	HUVECs	Regulates endothelial gene expression and angiogenic activity	[95]
MANTIS	↓	Rats	MCT induced	BRG1	PAECs	Promotes apoptosis and accelerates angiogenesis	[96]

MIAT, myocardial infarction-associated transcripts; GAS5, growth arrest-specific 5; MALAT1, metastasis-associated lungadenocarcinoma transcript 1; GATA6-AS, GATA6 antisense RNA 1 ELK1, ETS like-1 protein; EPC, endothelial progenitor cells; EndMT, endothelial-to-mesenchymal transition; PAECs, pulmonary arterial endothelial cells; HUVECs, human umbilical vein endothelial cells; Nrf2, nuclear factor erythroid 2-related factor 2; NAT8L, N-acetyltransferase-8-like protein; TGFBR2, transforming growth factor-beta receptor 2; Smad3, Smad family member 3; LOXL2, lysyl oxidase-like protein 2; BRG1, Brahma-related gene 1; MCT, monocrotaline; SP, spermidine; TGF-β1, transforming growth factor β1; ECs, endothelial cells; ${\mathrm{EH}}_{\text{ITSN}}$, intersectin-1s protein fragment with proliferative potential; ELK1, ETS-like transcription factor; HPH, hypoxic pulmonary hypertension.

#### 3.2.1 Myocardial Infarction-Associated Transcripts (MIAT)

MIAT was found to stimulate oxidative stress in an HPH model by sponging miR-29a-5p and inhibiting the nuclear factor erythroid 2-related factor 2 (Nrf2) pathway. MIAT was upregulated but miR-29a-5p was downregulated *in vitro* and *in vivo* model of HPH in rats. Knockdown of MIAT suppressed hypoxia-induced increases in cell viability, migratory capacity, and oxidative stress in hPAECs. miR-29a-5p is a target gene of MIAT, and silencing of miR-29a-5p partially attenuated the effects of MIAT on the hypoxia-induced proliferation and migration in hPAECs. In addition, previous studies have shown that overproduction of reactive oxygen species leads to organelle dysfunction, which induces HPH. Oxidative stress activates specific downstream signaling pathways that contribute to the activation of the Nrf2 pathway, and MIAT knockdown significantly prevents oxidative stress in HPH patients. Thus, MIAT, a novel lncRNA, could exacerbates oxidative stress in HPH models by sponging miR29a-5p and inhibiting the Nrf2 pathway [91].

#### 3.2.2 LncRNA Growth Arrest-Specific Transcript 5 (GAS5)

GAS5 promotes spermidine (SP)-induced autophagy through the miRNA-31-5p/N-acetyltransferase-8-like protein (NAT8L) axis in CTEPH patients. SP is a naturally occurring polyamine that acts as an autophagy enhancer [99], and endogenous SP dose dependently promotes autophagy in PAECs from patients with CTEPH. In addition, in previous studies, GAS5 was shown to play a role in various diseases, such as diabetic cardiomyopathy [100], atherosclerosis [101], osteoarthritis [102] and allergic rhinitis [103], by regulating autophagy. Wu *et al*. [92] collected PAECs from CTEPH patients and rat models and found that SP-induced autophagy interacted with GAS5 in PAECs. In addition, in PAECs, SP treatment reduced the expression level of miRNA-31-5p, which can induce endothelial dysfunction, and vascular remodeling. GAS5 facilitated SP-induced autophagy in PAECs by sponging miRNA-31-5p. Thus, miRNA-31-5p is an important regulator of PAEC autophagy, and NAT8L, a downstream target of miRNA-31-5p, is also involved in autophagy in PAECs. Taken together, these findings indicate that sp-induced autophagy *in vitro* and *in vivo* can be promoted by GAS5 through the miRNA-31-5p/NAT8L signaling pathway.

#### 3.2.3 LncRNA AERRIE (LINC01013)

Through EndMT, endothelial cells can develop a mesenchymal phenotype, a process that can occur during embryonic development or under pathological conditions [104]. During EndMT, vessels lose the capacity to maintain vascular homeostasis, which leads to the eventual development of atherosclerosis, PH, or fibrosis [105]. The expression of the lncRNA AERRI is upregulated in EndMT, however, silencing of AERRIE does not reverse EndMT, and high expression of AERRIE does not lead to EndMT or morphological or functional changes in the endothelium. Furthermore, JMJD2B acts as a regulator of EndMT, and was found to regulate AERRIE and, subsequently, SULF1; however, AERRIE was only partially required for SULF1 expression, and silencing of AERRIE exerted a regulatory effect on SULF1 expression but not on the expression of endothelial and mesenchymal markers. This pattern suggests that although AERRIE is a newly discovered EndMT factor, its regulation of mesenchymal markers and transcription factors is limited, and its specific mechanism of action remains to be elucidated [93].

#### 3.2.4 LncRNA Metastasis-Associated Lung Adenocarcinoma Transcript 1 (LncRNA MALAT1)

Circulating endothelial progenitor cells (EPCs) can differentiate into vascular endothelial cells and can also undergo TGF-β1-dependent EndMT, which plays an important role in pathological conditions in various organs, such as the heart and lung [106]. EndMT can be blocked by certain lncRNAs. For example, the expression of the lncRNA MALAT1, a metastasis- and cancer-promoting lncRNA, is regulated to reduce EndMT progression through miR-145, which is a well-characterized tumor suppressor that inhibits Smad3-mediated EndMT in cancer cells [107]. EndMT in TGF-β1-induced EPCs is accompanied by upregulation of MALAT1 and downregulation of miR-145. In these cells, MALAT1 and miR-145 directly bind and inhibit one another. MiR-145 specifically targets TGFBR2 and SMAD3 to prevent TGF-1-induced EndMT. Thus, MALAT1 regulates TGFBR2 and Smad3 through miR-145 to modulate TGF-β1-induced EndMT in EPCs. These findings imply that the MALAT1-miR-145-TGFBR2/Smad3 signaling pathway is critical for TGF-1-induced EndMT. However, a relative limitation of the above study is that whether this signaling axis plays a role during EndMT in PH was not determined and needs further exploration [94].

#### 3.2.5 LncRNA GATA6 Antisense RNA 1 (LncRNA GATA6-AS) 

The lncRNA GATA6-AS is upregulated in hypoxic endothelial cells, and silencing of GATA6-AS attenuates TGF-β2-induced EndMT and promotes blood vessel formation in mice *in vitro*. Mechanistically, GATA 6-AS can interact with and inhibit the function of the epigenetic regulator lysine oxidase (LOXL) 2, and it was also found to induce trimethylation of H3K4. The catalytic activity of LOXL2 is associated with transcriptional regulation via H3K4me3 deamination; namely, LOXL2 catalyzes the oxidative deamination of H3K4me3 on chromatin, a process that is negatively regulated by the hypoxia-induced nuclear lncRNA GATA6-AS, thereby controlling endothelial cell function *in vitro* and *in vivo* via a mechanism linked to the epigenetic regulation of endothelial gene expression [95].

#### 3.2.6 LncRNA MANTIS

Among the epigenetically regulated endothelial lncRNAs, lncRNA n342419, also called MANTIS, is the most strongly regulated and is regulated by the histone demethylase JARID1B. MANTIS is downregulated in iPAH patients and in rats receiving MCT treatment, and low MANTIS expression impairs the repair capacity of PAECs, thereby perpetuating vascular remodeling. CRISPR/Cas9-mediated loss or siRNA- or GapmeR-mediated silencing of MANTIS was found to inhibit the angiogenic sprouting and arrangement of endothelial cells. Mechanistically, MANTIS interacts with Brahma-related gene 1 (BRG1), a catalytic subunit of the evolutionarily highly conserved ATP-dependent SWI/SNF chromatin remodeling complex. BAF155 stimulates the remodeling activity of BRG1, and MANTIS maintains the ATPase activity of BRG1 via Brahma-related gene 1-associated factor 155 (BAF155) to remodel the nucleosome. In addition, MANTIS was found to target the angiogenesis-related endothelium genes SOX 18, SMAD 6, and COUP-TFII. The transcription of key endothelial genes such as SOX 18, SMAD 6 and COUP-TFII is regulated by ensuring efficient binding of the RNA polymerase II machinery, and MANTIS and BRG1 promote RNA polymerase II binding by reducing heterochromatin condensation to promote endothelial angiogenesis. In conclusion, MANTIS mediates and directs the effective transcription of critical endothelial genes by acting as a scaffold lncRNA in the chromatin remodeling complex and thus plays an important and unique role in endothelial cell function [96].

### 3.3 LncRNAs and Fibroblast in PH

The vascular adventitia is composed mainly of fibroblasts [108]. Fibroblasts are key regulators of vessel wall function in pulmonary and somatic circulation. During hypoxia, fibroblasts are activated and undergo phenotypic changes, including increased proliferation, differentiation, and contraction and upregulate of extracellular matrix proteins, and can also stimulate the release of inflammatory factors [109]. Vascular remodeling in PH is an important factor mediating the increase in PVR, and fibroblast overproliferation leads to thickening of the vascular adventitia [110], in addition to deposition or crosslinking of extracellular matrix components (i.e., collagen), which in turn contributes to thickening of the vascular wall, extension of vascular muscle into normal nonmuscular arterioles, and vascular stiffness [111, 112, 113]. An increasing number of studies have shown that lncRNAs are involved in fibrosis in various organs, including the liver, heart, and lung [21], and in PH, lncRNAs are involved in the regulation of fibrosis and pulmonary vascular remodeling mainly by regulating the phenotype of fibroblasts.

#### 3.3.1 LncRNA-LNC_000113

Activation of PAFs is involved in pulmonary artery remodeling in PAH. Many studies have shown that lncRNAs may play fibrotic roles in diverse diseases [21]. Indeed, a novel lncRNA, LNC_000113, was identified as an activator of PAF in PH model rats by Luo *et al*. [114] Galectin-3 is a potent activator of pulmonary adventitial remodeling, which promotes fibrotic proliferation of PAFs. RNA-seq analysis revealed that the expression of the lncRNA LNC_000113 was significantly greater in Galectin-3-treated PAFs. Furthermore, the expression of the lncRNA LNC_000113 gradually increased in the lungs of MCT model rats. The results of loss-of-function experiments indicated that Galectin-3-induced activation of PAFs requires the lncRNA LNC_000113. The potential mechanism involves the regulation of the PTEN/Akt/FoxO 1 pathway. These findings suggest a profibrotic function of the lncRNA LNC_000113 in pulmonary artery remodeling. Modulation of lncRNA LNC_000113 expression could be used as an antifibrotic therapeutic strategy to attenuate pulmonary artery remodeling in PH.

#### 3.3.2 Hyaluronan Synthase 2 Antisense 1 (HAS2-AS1)

HAS2-AS1 is a natural antisense RNA derived from the gene encoding hyaluronic acid synthetase 2 (HAS2), a major component of most extracellular matrices, and its overexpression reverses the suppression of cellular EndMT and migration [115]. The proliferation and migration capacities of human fetal lung fibroblast 1 (HFL-1) cells were found to be significantly increased under hypoxic conditions, and the expression of HAS2-AS1 and HAS2 was detected under hypoxic conditions. More importantly, cytokines associated with inflammation, including IL-6, IL-1β and TNF-α, are strongly expressed under hypoxia. Mechanistically, hypoxia-induced inflammation is largely influenced by C/EBP, whose expression is increased when hypoxia occurs; moreover, through its transcription factor activity, C/EBPβ regulates HAS2-AS1 expression by binding to its promoter region. Downregulation of C/EBPβ was also found to decrease HAS2-AS1 expression, resulting in decreased proliferation and migration of HFL-1 cells, accompanied by decreased expression of TNF-α, IL-1β and IL-6. In conclusion, hypoxic conditions can promote the expression of the inflammation-related transcription factor C/EBPβ and the lncRNA HAS2-AS1, which are associated with the extracellular matrix, and the transcription factor C/EBPβ promotes the migration, proliferation and inflammation of HFL-1 cells by activating lncRNA HAS2-AS1 expression in hypoxia [116].

### 3.4 LncRNAs that Participate in the Inflammatory Immune Response

LncRNAs are also widely involved in inflammatory and immune responses; in fact, inflammatory processes are associated with metabolic changes in vascular and inflammatory cells, and several lncRNAs have been demonstrated to participate in vascular inflammatory reactions [117, 118].

#### 3.4.1 Nuclear Factor-kappaB Interacting LncRNA (NKILA)

The lncRNA MALAT1 upregulates the glucose-induced inflammatory mediators TNF-α and IL-3 by activating serum amyloid antigen 6 in HUVECs [119]. Furthermore, MALAT1 can rescue the TGF-β type II receptor from posttranscriptional repression by sponging miR-145 in EPCs [120]. NKILA is a cytoplasmic lncRNA that can regulate endothelial inflammation by controlling the NF-κ b/klf 4 positive feedback loop, acting as a critical regulator to protect against the development of endothelial inflammatory lesions and related vascular diseases [121]. In addition, the lncRNA Giver is induced by the novel molecule angiotensin II, which is induced by proinflammatory cytokines, through the upregulation of IL-6, CCL-2 and TNF to increase oxidative stress, the inflammatory response and proliferation in vascular smooth muscle cells [122].

#### 3.4.2 LncRNA NONRATT009275.2

Patients with all types of PH and animal models both experience the development of perivascular inflammatory infiltrates. These cells include T and B lymphocytes, mast cells, dendritic cells, and macrophages [123]. Hou *et al*. [124] simultaneously examined the expression profiles of lncRNAs and mRNAs in the lungs of rats with MCT-induced PH by high-throughput sequencing and found that 559 lncRNAs and 691 mRNAs were differentially expressed in the lungs. These aberrantly regulated lncRNAs and mRNAs were involved in important biological processes and pathways related to PH, and inflammation and the immune response were the pathways exhibiting major enrichment. One of the newly identified PH-associated lncRNAs, NONRATT009275.2, promotes macrophage polarization toward the M2 phenotype and thus participates in the inflammatory immune response.

### 3.5 LncRNAs and Sex Bias in PH

PH is a disease that favors women, with about four times as many women suffering from PH as men, but with greater severity in men. The underlying causes of this sexual dimorphism have been investigated for many years and many hypotheses have been proposed, usually involving alterations in sex hormones, with the Y-chromosome genes protective effects, genetics and the immune system also likely to play a role [125, 126]. Studies have shown that lncRNAs also contribute to gender bias in PH. Qin *et al*. [97] found that the proliferative potential of ECs is gender dimorphic, with lncRNA xist involved in this process. The specific mechanism involves an endocytosis protein, Intersectin-1s (ITSN), which is important for the dysfunctional molecular phenotype of ECs in PH. Granzyme B cleaves ITSN to create the N-terminal ${\mathrm{EH}}_{\text{ITSN}}$ protein fragment during PH-related inflammation. The protein fragment known as ${\mathrm{EH}}_{\text{ITSN}}$ is reactive and has the ability to promote the proliferation of ECs by continuously activating the p38-ELK1-c-Fos signaling axis. It is observed that the expression of ${\mathrm{EH}}_{\text{ITSN}}$ is more responsive in female cells compared to male cells, possibly because in female PAECs, Xist is significantly upregulated in terms of both expression and activity. This, in turn, significantly upregulated the X-linked gene ELK1 and suppressed the expression of KLF2, which encodes a key transcription factor that regulates the signaling pathway for EC dysfunction characteristic of PH. These molecular events explain the gender dimorphism in the proliferative response of PAECs and the imbalance in the PH sex ratio that may result from the upregulation of Xist. More significantly, Xist has a role in dose compensation and sex determination in mammals. In females, an extra X chromosome causes an imbalance in the expression of X-linked genes if dosage compensation is not applied. Randomized X chromosome inactivation (XCI) generates dosage compensation in females, and Xist is believed to be essential for randomized XCI [98]. In the lungs of female ${\mathrm{EH}}_{\text{ITSN}}$-KO^ITSN+/-^ mice, Xist is markedly overexpressed, leading to sex-specific regulation of the Elk1 transcription factor, cell cycle protein A1, Elk1 target proteins, and a cell cycle regulatory protein. Additionally, the XCI-linked PH gene is also regulated. These molecular occurrences were more prominent in female than in male mice models, and lung specimens from female patients with PH also demonstrated sex-specific modulation of Elk1 and cell cycle protein A1 expression. These evidences strongly suggest that lncRNA-Xist upregulation explains the gender dimorphism in the proliferation of female PAECs in PH [127]. Interestingly, Qin *et al*.’s [98] ${\mathrm{EH}}_{\text{ITSN}}$-KO^ITSN+/-^ mice model of plexiform arteriopathy showed a ~22% increase in muscularization in small and medium-sized pulmonary arteries, with females showing a substantially higher degree of medial thickening (~2 orders of magnitude higher). This implies that sexual dimorphism in PH is present in both ECs and smooth muscle cell (SMC) proliferation, and that Xist upregulation in ECs is the cause of this sexual dimorphism in SMC proliferation. Apparently, SMCs also have an effect on PH gender bias, as shown by Yang *et al*. [128, 129] who found that sex bias in chronic hypoxia-induced PH in mice was removed by smooth muscle-specific BCL6+/-knockdown or deletion of STAT5a/b, in addition, there are studies related to Xist and fibroblasts in other sex biased diseases such as systemic lupus erythematosus and rheumatoid arthritis [130], perhaps there is also some connection between Xist and the sexual dimorphism of fibroblasts in PH. Unfortunately, studies on the direct effects of Xist on SMCs and vascular fibroblasts have not been published, and direct studies of lncRNA expression and how its enhanced expression affects SMCs and vascular fibroblasts remain a major need in PH sex bias.

### 3.6 LncRNAs and Right Ventricular Dysfunction

In individuals with PH, the right ventricle’s (RV) capacity to respond to pressure overload is a key factor in determining functional status and prognosis. Previously, a study by Omura *et al*. [131] was the first to identify a the direct role of the lncRNA H19 in right ventricular failure in patients with PH and provided evidence that H19 impairs right ventricular function in patients with PH. H19 was overexpressed in the decompensated RVs of patients with PH and in PAB- and MCT-induced rat models of PH, and the level of H19 expression was significantly correlated with the severity of right ventricular remodeling and biochemical and histological features of deteriorating cardiac function. GapmeR-mediated downregulation of H19 induced upregulation of the E2F1/EZH2 axis, which ameliorated the features of right ventricular hypertrophy, capillary thinning, and fibrosis in PH without affecting pulmonary vascular remodeling. Silencing of H19 suppressed but overexpressing of H19 promoted phenylephrine-induced cardiomyocyte hypertrophy, suggesting that H19-mediated downregulation of the E2F1/EZH2 axis is involved in right ventricular hypertrophy, capillary thinning, and fibrosis. Furthermore, it was found that MALAT1 inhibition mediated by intraperitoneal injection of the locked nucleic acid (LNA) GapmeR decreased the heart weight of mice with established hypoxia-induced PH, and silencing MALAT1 with GapmeRs reduced cardiac hypertrophy in mice with hypoxia-induced PH. However, in a PH mouse model, right ventricular systolic pressure (RVSP) was not changed by MALAT1 inhibition, and the cause of this phenomenon was not clear; thus, the specific mechanism by which MALAT1 participates in cardiac remodeling remains to be explored [25].

### 3.7 LncRNA and Different Types of PH

Based on blood flow dynamics and pathological manifestations, PH is generally divided into five subgroups, pulmonary arterial hypertension (PAH), PH due to left heart disease, PH due to lung diseases and/or hypoxia, chronic thromboembolic PH, and PH with unclear multifactorial mechanisms [132]. An animal or cellular model of hypoxia is the basis for the majority of the lncRNAs discussed previously in the article. Furthermore, utilizing the tissues of PAH patients, other lncRNAs, such as Lnc-Ang362, PAXIP1-AS1, MEG3, and MALAT1, have been investigated [133]. One need to supplement is idiopathic PAH (IPAH), IPAH patients are essentially PAH patients, and research has revealed a close relationship between lncRNAs and iPAH. Major dysregulated pathways were found, and important lncRNAs involved in PAH were highlighted by Wang *et al*.’s [134] systematic data analysis of lncRNAs in IPAH peripheral blood monocytes (PBMCs). Using differential expression and further studies, the researchers found 18 pairs of lncRNAs and PPI modules. These included four PPI modules (OAS 1, CXCL10, STAT 1, and TLR 4) and seven lncRNAs (LOC643888, LOC554206, IL8RBP, LOC642897, SLC6A10P, RPL23AP7, and LOC400759). These lncRNAs are intimately associated to the PPI module, which is involved in proinflammatory pathways, chemokine signaling, and NOD-like receptor signaling. Taken together, these findings indicate that these lncRNAs contribute significantly to the pathophysiology of IPAH through proinflammatory pathways. In addition, research on lncRNAs in other PH subgroups has been essentially absent. The analysis reasons may be as follows: first, PH patients in other subgroups are less common than PAH patients, and it is more challenging to obtain clinical study samples; second, the causes of PH formation in other subgroups are multifaceted and intricate; and, finally, there is greater interference with the indicators affecting the diseases to be included.

### 3.8 Clinical Significance of lncRNAs in PH

Through several molecular pathways, lncRNAs can control the expression of protein-coding genes; for example, lncRNAs can participate in epigenetic regulation, and target gene control via transcriptional and posttranscriptional mechanisms is crucial for many physiological and pathological processes, such as speciation, development and aging of organisms; maintenance of cellular homeostasis; metabolism; and progression of disease development. Therefore, lncRNAs have very broad application prospects in the diagnosis and treatment of diseases. Various lncRNAs are involved in the development of PH, and these lncRNAs may not only be used as molecular diagnostic markers for this disease but also be important in the prognostic assessment of patients with PH and in targeted therapy for PH. However, several limitations remain. First, because lncRNA expression is typically low and is tissue and cell specific, delivering RNA inhibitors or viral vectors to certain cell layers can be challenging. Thus it is difficult to develop drugs targeting lncRNAs [135]. Second, lncRNAs undergo extensive alternative splicing [136]. Thus, most lncRNAs are produced as many isoforms, which complicates our knowledge of their localization and function while also underlining the possibility of their varied subcellular localization and the unique functional significance of their subcellular localization. Many studies have begun to elucidate this essential yet almost neglected facet of lncRNA biology; however, research on the location and purpose of particular lncRNA isoforms is scarce [137]. Finally, most studies on the use of lncRNAs as therapeutic targets have used early-stage cellular and animal models, and it is clear that findings in animals cannot be directly translated to humans. Moreover, stimulating cells by inducing PH phenotypes in artificial environments cannot fully reproduce the complex mechanisms of PH induction in humans. Furthermore, in cell-based assays, therapeutic substances can be delivered directly into the cells. Therefore, the development of effective drug delivery systems is crucial [66]. However, targeting lncRNAs is undeniably a potentially promising therapeutic strategy. Therefore, we still need intensive research and clinical trials are needed to advance the development of novel PH treatments on which innovative diagnostic and therapeutic protocols may be based and incorporated clinically.

## 4. Conclusions

To sum up, lncRNAs play a complex and critical role in the pathogenesis of PH. They play a crucial role in the progression and development of the disease through their multifaceted roles in regulating gene expression, intercellular communication, and vascular remodeling. However, despite the remarkable progress in recent years, the functional networks and regulatory pathways of lncRNAs in pulmonary arterial hypertension (PAH) still need to be further explored in depth. Future studies should focus on the following directions: first, identify more pulmonary arterial hypertension-associated lncRNAs through large-scale genomic and transcriptomic analyses and explore their functional specificities and regulatory modes. Second, resolve the interactions of lncRNAs with classical molecular pathways, such as signaling pathways and epigenetic regulation. Finally, to translate basic research findings into innovative diagnostics and therapies in the clinic, focusing on the specificity and deliverability of molecular drugs, carefully evaluating appropriate clinical outcome metrics, and developing targeted lncRNA drugs or biomarkers with the aim of early diagnosis and personalized treatment. Such studies will not only reveal new biological mechanisms of pulmonary hypertension, but also provide potential therapeutic targets for this disease, opening up new possibilities for improving patient prognosis.
